# Properties of Injectable Apatite-Forming Premixed Cements

**DOI:** 10.6028/jres.115.017

**Published:** 2010-08-01

**Authors:** Yashushi Shimada, Laurence C. Chow, Shozo Takagi, Junji Tagami

**Affiliations:** Tokyo Medical and Dental University, Tokyo, Japan; American Dental Association Foundation, Paffenbarger Research Center, National Institute of Standarsds and Technology, Gaithersburg, Md 20899, U.S.A.; Tokyo Medical and Dental University, Tokyo, Japan

**Keywords:** bone graft, crystallinity, dicalcium phosphate anhydrous, dual-paste premixed calcium phosphate cement, tetracalcium phosphate

## Abstract

Previous studies reported premixed calcium phosphate cements (CPCs) that were stable in the package and form hydroxyapatite (HA) as the product after exposure to an aqueous environment. These cements had setting times of greater than 60 min, which are too long to be useful for some clinical applications. The present study investigated properties of fast-setting HA-forming premixed CPCs that initially consisted of two separate premixed pastes: (1) finely ground (1.0 μm in median size) dicalcium phosphate anhydrous (DCPA) mixed with an aqueous NaH_2_PO_4_ solution, 1.5 mol/L or 3.0 mol/L in concentration, and (2) tetracalcium phosphate consisting of combinations of particles of two different size distributions, 5 μm (TTCP5) and 17 μm (TTCP17) in median size, mixed with glycerin. Equal volume of Pastes 1 and 2 were injected with the use of atwo-barrel syringe fitted with a static mixer into sample molds. The molar Ca/P ratio of combined paste was approximately 1.5. Cements were characterized in terms of setting time (Gilmore needle), diametral tensile strength (DTS), and phase composition (powder x-ray diffraction, XRD). Setting times were found to range from (4.3 ± 0.6 to 68 ± 3) min (mean ± sd; n = 3), and 1-d and 7-d DTS values were from (0.89 ± 0.08 to 2.44 ± 0.16) MPa (mean ± sd; n = 5). Both the NaH_2_PO_4_ concentration and TTCP particle size distribution had significant (p < 0.01) effects on setting time and DTS. Powder XRD analysis showed that low crystallinity HA and unreacted DCPA were present in the 1-day specimens, and the extent of HA formation increased with increasing amount of TTCP5 in the TTCP paste. Conclusion: Injectable HA-forming premixed CPCs with setting times from 4 to 70 min can be prepared by using DCPA and TTCP as the ingredients. Compared to the conventional powder liquid cements, these premixed CPCs have the advantages of being easy to use and having a range of hardening times.

## 1. Introduction

Since their first development in the 1980’s, calcium phosphate cements (CPC) have attracted considerable attention because these materials set like cements and form nanocrystalline hydroxyapatite (HA) [1] or microcrystalline dicalcium phosphate dihydrate (DCPD) [2] as the major product. The nanocrystalline HA from CPC is formed in an aqueous environment at body temperatures, hence it is more similar to biological apatites than sintered HA formed at high temperatures [3,4]. CPC is bioactive, non-cytotoxic and osteoconductive. More importantly, it is gradually replaced by new bone without volume loss [5–7]. Due to its excellent osteoconductivity and bone replacement capability, CPC is highly promising for a range of clinical applications [3,4]. The properties of the various CPC materials currently in clinical use have been extensively reviewed in several publications [8–10].

Nearly all of the presently available CPCs are in the form of a powder and a liquid that are mixed immediately before use. Under clinical situations, the ability to properly mix the cement and then place it in the defect within the prescribed time is a crucial factor in achieving optimum results. Thus, it would be desirable to have a premixed cement paste, prepared in advance using a well controlled process, that is stable in the package and hardens only after being placed in the defect. Previous studies [11–13] evaluated such a cement prepared by combining CPC powders with anhydrous glycerin, a non-aqueous but water miscible liquid. The absence of water kept the premixed paste unreacted and soft until it was placed in the defect, where diffusion of water from the tissues into the CPC would cause the cement to harden. While this premixed CPC has the advantage of infinite work time, especially desirable when used as an injectable CPC, it hardened too slowly (60 min) for most clinical applications. Several rapid hardening premixed CPCs were subsequently developed that have setting times below 10 min, and had non-cytotoxicity matching the conventional CPC [12]. However, these formulations have a relatively short shelf life because of the high reactivity with moisture. Further, the interior of a large implant would not harden for a long time due to slow water diffusion.

Recently, Lemaitre et al., [14] reported a two-paste brushite-forming CPC. In this cement system, the individual cement components, *β*-tricalcium phosphate (*β*-TCP) and monocalcium phosphate monohydrate (MCPM) are sufficiently stable when present in separate aqueous pastes. Combination of the two pastes led to rapid cement setting. Since HA-forming cements are the most commonly used CPCs in humans, we report here a dual-paste HA-forming CPC. The mechanical properties and HA conversion of this new dual-paste premixed CPC system were evaluated in this study.

## 2. Materials and Methods

### 2.1 Preparation of TTCP and DCPA Powders

Tetracalcium phosphate (TTCP), Ca_4_(PO_4_)_2_O, was prepared following a method previously described [15]. Briefly, commercially obtained dicalcium phosphate anhydrous (DCPA), CaHPO_4_, (J. T. Baker Chemical Co., Phillipsburg, NJ)^1^ and CaCO_3_ (J. T. Baker Chemical Co.) were thoroughly mixed and heated at 1500 °C for 6 h in a furnace (Model 51333, Lindburg, Watertown, WI) and quenched at room temperature in a desiccator. The solid was first crushed by mortar and pestle, and then dry ground in a ball mill (Retsch PM4, Brinkman, NY) to obtain the desired median particle size of 17.0 μm (TTCP17). TTCP17 was further ground in a ball mill in water-free cyclohexane for 24 h to obtain a medium of particle size of 5.0 μm (ground TTCP5). The commercial DCPA was also ground for 24 h in the ball mill in 95 % ethanol to obtain a median particle size of 1.0 μm.

### 2.2 Experimental Design

This investigation was designed to examine the effects of the following two independent variables on the setting time, porosity, diametral tensile strength (DTS), and HA formation of the experimental cements:

1. The concentration of NaH_2_PO_4_ in the cement liquid. The NaH_2_PO_4_ was supplied in the DCPA-containing paste.
2. The TTCP particle size.

### 2.3 Preparation of Pre-Mixed Cement Pastes

The cements consisted of two premixed pastes: (1) DCPA-containing paste and (2) TTCP-containing paste. These pastes were prepared in advance as described below.

1. The DCPA-containing pastes were prepared by mixing DCPA with either a 1.5 mol/l or 3.0 mol/l NaH_2_PO_4_ solution at powder/liquid (P/L) mass ratio of 1.43. These pastes were designated as DCPA(1.5) and DCPA(3), respectively. The phosphate solutions were prepared by dissolving reagent grade NaH_2_PO_4_ (Spectrum Chemical Mfg. Corp., Gardena CA) in distilled water.
2. TTCP-containing pastes were prepared by combining TTCP powders of various particle sizes with water at a P/L mass ratio of 1.43. To investigate the effects of TTCP particle size, four TTCP-containing pastes were prepared using TTCP powders consisting of : (a) TTCP17 only, designated as 100 % large TTCP, or TTCP (100L), (b) 25 % TTCP17 and 75 % TTCP5, designated as 25 % large and 75 % small TTCP, or TTCP(25L75S), (c) 50 % TTCP17 and 50 % TTCP5, designated TTCP(50L50S), and (d) TTCP5 only, designated as TTCP(100S). Beginning from paste (b), the fraction of TTCP5 was doubled in each step.

The aqueous solution present in both the DCPA and TTCP-containing pastes also contained 1 wt % hydroxypropyl methylcellulose (HPMC), a derivative of cellulose and one of the most commonly used ingredients in food and medicinal formulations, as a gelling and lubricating agent [16]. The P/L ratio of 1.43 was chosen to produce pastes that exhibited workable consistencies.

In the study, each of the four TTCP pastes described above was mixed with each of the two DCPA pastes at a mass ratio of 1:1. At the P/L ratio of 1.43, the Ca/P molar ratio of the combined pastes were calculated to be 1.32 and 1.22 when the TTCP paste was combined with the DCPA1.5 and DCPA3 pastes, respectively. The phosphate concentration in the cement liquid and the TTCP particle size were the two independent variables being investigated.

### 2.4 Hardening Time

Hardening times of the CPC samples were estimated by both the results of Gilmore needle method test and washout resistance test. Gilmore needle method test was performed using a needle with a tip diameter of 1.06 mm loaded with 453.5 g. The two pastes of the desired ratio were hand mixed using a spatula on a glass slab for approximately 10 s, placed into a mold (approximately 6 mm in diameter and 3 mm in depth) sandwiched between two plastic plates, and stored in a humidor with 100 % relative humidity at 37 °C. In this method, the cement was considered set when the needle with the load failed to make a perceptible indentation on the surface.

### 2.5 Diametral Tensile Strength (DTS) Measurement

DTS specimens for the mixed dual paste CPCs were prepared with the use of a previously described method [17]. Pre-mixed TTCP paste and DCPA paste were mixed and packed into a stainless steel mold, formed by two rods (6 mm diameter) in a cylindrical cavity, with ≈2MPa of applied pressure, which was placed in an incubator kept at 37 °C and 100% humidity for 4 h. The specimens were then removed from the molds and immersed in 30 ml of a physiological-like solution (PLS) [11], containing 1.15 mmol/L Ca, 1.2 mmol/L P, 133 mmol/L NaCl, 50 mmol/L HEPES buffer and with pH adjusted to 7.4, at 37 °C for 20 h (1-day group) or 1 week (1-week group). For the 1-week group, the PLS was changed daily. After the immersion in PLS, the diameter and length of each specimen was measured with a micrometer and the samples were placed on a universal testing machine (United Calibration Corp, Garden Grove, CA) for DTS measurement. The specimen was stressed between steel platens that were covered with one thickness of wet filter paper and crushed at a loading rate of 10 mm/min. The force at failure was recorded and converted to a stress in MPa unit. DTS value was the average value obtained from five specimens.

### 2.6 Porosity Ratio Within the Cement

Crushed DTS specimens were collected and heated at 110 °C for 24 h. The specimen density was calculated from the dried weight and sample dimensions. The porosity of the specimen was calculated by comparing the specimen density with the crystal density of hydroxyapatite [18].

### 2.7 Conversion to Hydroxyapatite (HA)

The conversion of CPC starting materials to HA was assessed by powder x-ray diffraction (XRD) analyses. After the DTS test, the specimens of 1day and 1week groups were dehydrated in 100 % ethanol for 1 h and dried in the desiccators for 3 days to stop the process of further CPC conversion to HA. The dried specimens were ground to fine powders with a mortar and pestle, and characterized by XRD. The XRD patterns of the specimens were recorded with the use of a vertically-mounted diffractometer system (D/MAX 2000, Rigaku, Danvers, MA) with graphite-monochromatized CuK*_α_* radiation (*λ* = 0.1540 nm) generated at 40 kV and 40 mA. The sample was scanned from 20 to 40 degrees 2θ in a continuous mode (2° 2θ min^−1^, time constant 2 s), and the peak intensities were recorded digitally. The relative peak intensities of the 0-1-3 (20 = 29.2°) and 0-4-0 (29.8°) reflections for TTCP, the 1-1-0 (26.6°) reflection for DCPA, and the 0-0-2 (25.9°) reflection for HA were used to approximate the HA formation.

### 2.8 Statistical Analysis

The porosity and DTS data were analyzed statistically using ANOVA and Newman-Keuls multiple comparisons at a significance level of p = 0.05. In this study, the standard deviation of each of the measured parameters was considered as the standard uncertainty for that variable.

## 3. Results

### 3.1 Hardening Time

Hardening times of the experimental CPCs (Table 1) varied widely, from approximately 4 min to 68 min. Two-way ANOVA showed that the effects on hardening time from both independent variables, the TTCP particle size and the phosphate concentration in the cement liquid, and their interaction were significant (p < 0.05). The hardening time decreased with decreasing TTCP size and with increasing phosphate concentration.

### 3.2 Porosity

Due to the low P/L (1.43) used for preparing the pastes, the set cement specimens had relatively high-porosities, ranging from approximately 50 vol % to 60 vol % (Table 2). For the 1-d samples, two-way ANOVA, with TTCP size and the cement liquid phosphate concentration as the independent variables, showed that both factors produced significant (p < 0.05) effects on porosity, whereas their interaction was not significant (p > 0.05). Multiple comparison of the marginal means showed that higher phosphate concentration led to lower porosity. On the other hand, with the exception of the two groups (TTCP100L and TTCP75L25S) with the largest TTCP particles, decreasing TTCP size led to higher porosity (Table 2).

For the 7-d samples (7-d immersion in PLS), two-way ANOVA show that both the TTCP size and the cement liquid phosphate concentration, as well as their interaction, produced significant (p < 0.05) effects on the porosity. However, unlike with the 1-d samples, TTCP size effect appeared to be different (Table 2) for the two phosphate groups (DCPA1.5 and DCPA3). In contrast, porosity still decreased increasing phosphate concentration as in the 1-d samples.

Three-way ANOVA of the combined 1-d and 7-d data showed that all three factors (TTCP size, phosphate concentration, and immersion time) and two of the three interactions (TTCP size-phosphate concentration and TTCP size-immersion time) produced significant (p < 0.05) effects on porosity. A noteworthy observation was that the porosity of the 7-d samples was higher than the corresponding values of the 1-d samples for the two groups prepared with the largest TTCP particles (TTCP100L and TTCP75L25S), whereas for the remaining two groups (TTCP25L75S) and TTCP100S), the porosity did not change with between the 1-d and 7-d samples.

### 3.3 DTS

The DTS of the 1-d samples ranged from approximately 0.9 MPa to 2.3 MPa (Table 3). These values are lower than previously reported DTS values for the TTCP+DCPA cements, most likely due to the low P/L (1.43) used, which led to higher porosities (Table 2).

Two-way ANOVA showed that both the TTCP size and cement liquid phosphate concentration, but not their interaction, produced significant effects on DTS. Multiple comparison of the marginal means indicated that all but the TTCP group with the smallest particles (TTCP100S) have similar DTS values, which are significantly (p < 0.05) higher than the DTS of the TTCP100S group. On the other hand, the DTS values were consistently lower for samples prepared with the higher phosphate concentration in the cement liquid (DCPA3).

The DTS of the 7-d samples ranged from approximately 0.9 MPa to 2.4 MPa, nearly the same as that of the 1-d samples. Two-way ANOVA showed that both the TTCP size and cement liquid phosphate concentration, as well as their interaction, produced significant effects on DTS. For the lower phosphate group (DCPA1.5), the DTS of the first three TTCP groups were not different and were significantly higher than that of the last TTCP group (TTCP100S), as observed in the 1-d samples. For the higher phosphate group (DCPA3), the DTS generally decreased with decreasing TTCP size. With the exception of TTCP100L, lower DTS values resulted with the use of higher cement liquid phosphate concentration.

Three-way ANOVA of the combined 1-d and 7-d samples showed that the TTCP particle size and phosphate concentration of the cement liquid, but not the PLS immersion time, produced significant effects on DTS. However, two-way interactions from TTCP particle size-phosphate concentration and from TTCP-particle size-immersion time also produced significant effects. A comparison of the 1-d and 7-d data showed that, with the exception of the TTCP100L group, DTS values were essentially unaffected by PLS immersion time. For the TTCP100L, DTS increased significantly with PLS immersion for both phosphate concentration groups.

### 3.4 Conversion to HA

**Fig. 1:**
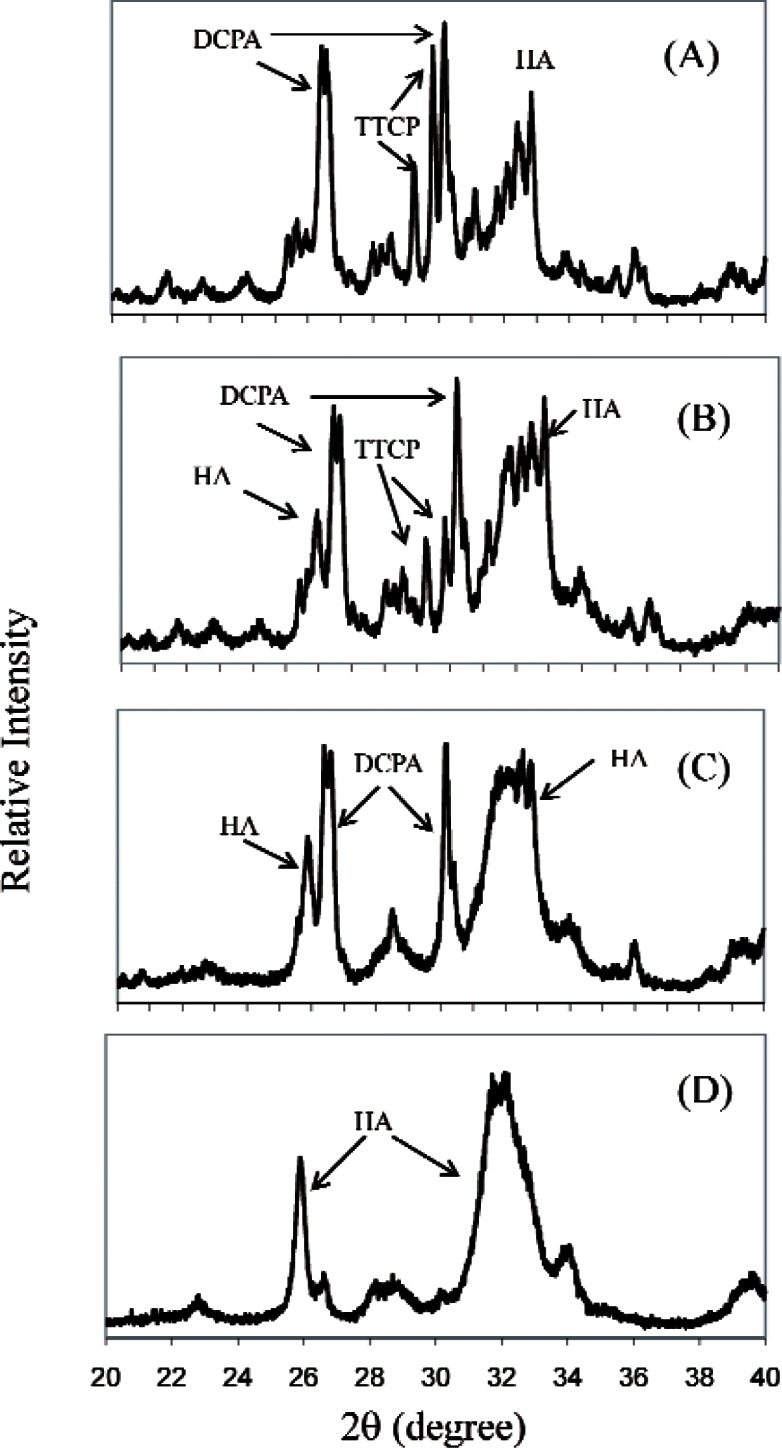
Powder x-ray diffraction (XRD) patterns of samples in PLS for 1 day and 7 days. (A) and (B) denote XRD patterns of samples prepared from pastes (TTCP100L + DCPA1.5) and incubated in PLS for 1 day and 7 days, respectively. (C) and (D) correspond to samples prepared from pastes (TTCP100S + DCPA1.5) and in PLS for 1 day and 7 days, respectively.

There were unreacted DCPA in all 1-d as well as 7-d samples (Fig. 1). This is likely because of the low overall Ca/P molar ratios (1.32 and 1.22) of cements, leading to the presence of an excessive amount of DCPA relative to the amount of TTCP available for forming HA as the product. A larger amount of unreacted DCPA was generally found in samples prepared using DCPA3, i.e., a higher phosphate concentration in the cement liquid, probably due to the even lower Ca/P ratio of 1.22. In many cases, the unreacted DCPA in the 7-d sample was lower than that in the corresponding 1-d sample. This suggests that DCPA continued to react during PLS incubation either by itself or with the unreacted TTCP.

Unreacted TTCP was also found in all 1-d samples, but TTCP was found in 7-d samples only in the cases when the TTCP paste used contained some coarse TTCP. This suggests that despite of a shortage of TTCP relative to the amount of DCPA present, the coarse TTCP was unable to be fully consumed in the reaction with DCPA to form HA either in the 1-d or 7-d time period.

## 4. Discussion

A wide range of setting times, from about 4 min to 68 min, was observed for this dual-paste premixed cement system. The setting time was found to depend most strongly on the TTCP particle size and then on the phosphate concentration of the cement liquid. The results suggest that cements with clinically required setting times can easily be formulated for this premixed cement system.

The highest DTS value obtained in the present study was 2.44 MPa, which is about 1/4 that of the conventional powder/liquid TTCP+DCPA cement. The low DTS values can be mostly attributed to the low P/L ratio of 1.43 used in this study, compared to P/L of 3 used in most previous studies, necessary to prepare freely injectable pastes. A secondary reason for the low DTS values is likely to be the low Ca/P ratio of the cements, i.e., 1.32 and 1.22, respectively, when the DCPA1.5 and DCPA3 pastes were used. Previous studies [19,20] showed that the DTS of the TTCP+DCPA cement with Ca/P ratio of 1.33 was about 7 MPa compared to about 10 MPa for cement with Ca/P of 1.67. Although CPCs with DTS below 2 MPa are being used successfully for certain clinical applications [21], the strength of the dual-paste premixed cements can be improved by increasing the P/L ratio while maintaining adequate flow properties for injection with the use of deflocculating agents such as sodium citrate [22]. The DTS can also be expected to increase by increasing the Ca/P ratio of the combined paste through the use of 2:1 rather than 1:1 mass ratio of the TTCP and DCPA pastes.

The crystallinity of the apatitic cement product in the cements that used the small TTCP, i.e., TTCP(100S), was significantly lower than that of cement that used coarse TTCP, TTCP(100L) (Fig. 1). This observation is consistent with previous findings for the conventional powder/liquid TTCP+DCPA cements [23]. This suggests that the crystallinity and possibly the *in vivo* resorption rate of the TTCP+DCPA CPCs can be controlled to a degree by the TTCP particle size.

A similar premixed cement consisted of two aqueous pastes was suggested by Lamaitre et al. [14]. The first paste contained TTCP and water, and the second paste contained DCPA, HA and an aqueous phosphoric acid solution. Because no experimental data were reported, it is not possible to compare the setting and strength properties of this cement with those of the cement investigated in the present study. An important question about Lamaitre’s and our dual-paste premixed cement is the stability and therefore the shelf life of the premixed pastes. In a preliminary study the premixed pastes were stored in separate containers, and were kept at 45 °C for 1 week. The setting times were then measured of the combined pastes consisting of all combinations of fresh and aged TTCP and DCPA pastes. The result showed that the DCPA paste was essentially unaffected by the aging, whereas the aged TTCP paste lost its reactivity, leading to excessively long setting times. The results suggest that it would be necessary to formulate TTCP paste in such a way that assures its stability over a sufficiently long time period.

## Figures and Tables

**Table 1:** Mean cement setting time as a function of TTCP particle size and phosphate concentration in the cement liquid

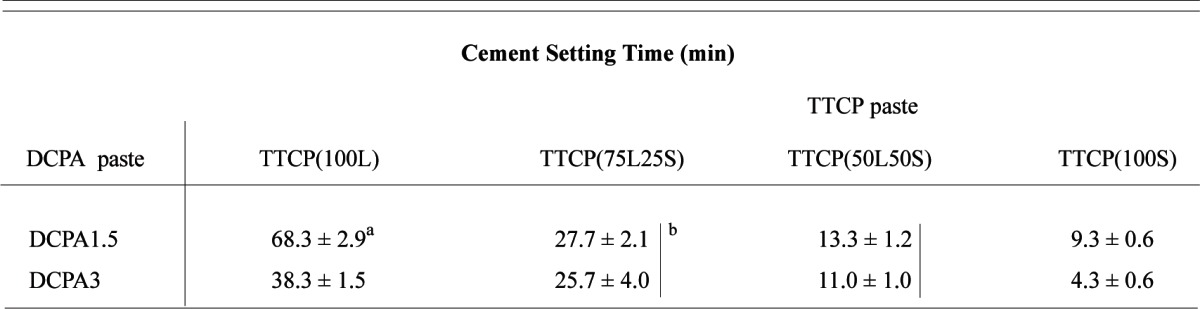

a Mean ± s.d. (n = 3)

b Values connected by a line are not significantly different (p > 0.05)

**Table 2:** Mean porosity of cements as a function of the TTCP particle size and phosphate concentration in the cement liquid, and the length of immersion time in PLS

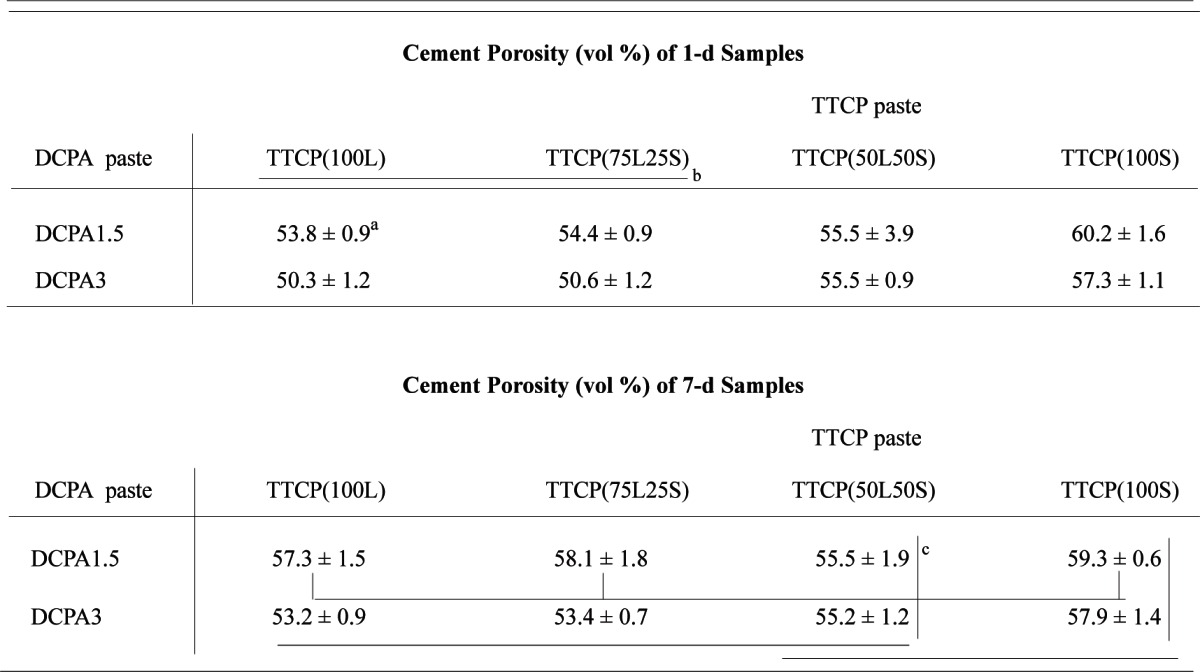

a Mean ± s.d. (n = 5)

b Groups connected by a line on the margins are not significantly different (p > 0.05)

c Groups connected by a line are not significantly different (p > 0.05)

**Table 3:** Mean DTS of cements as a function of the TTCP particle size, phosphate concentration in the cement liquid, and length of immersion in PLS

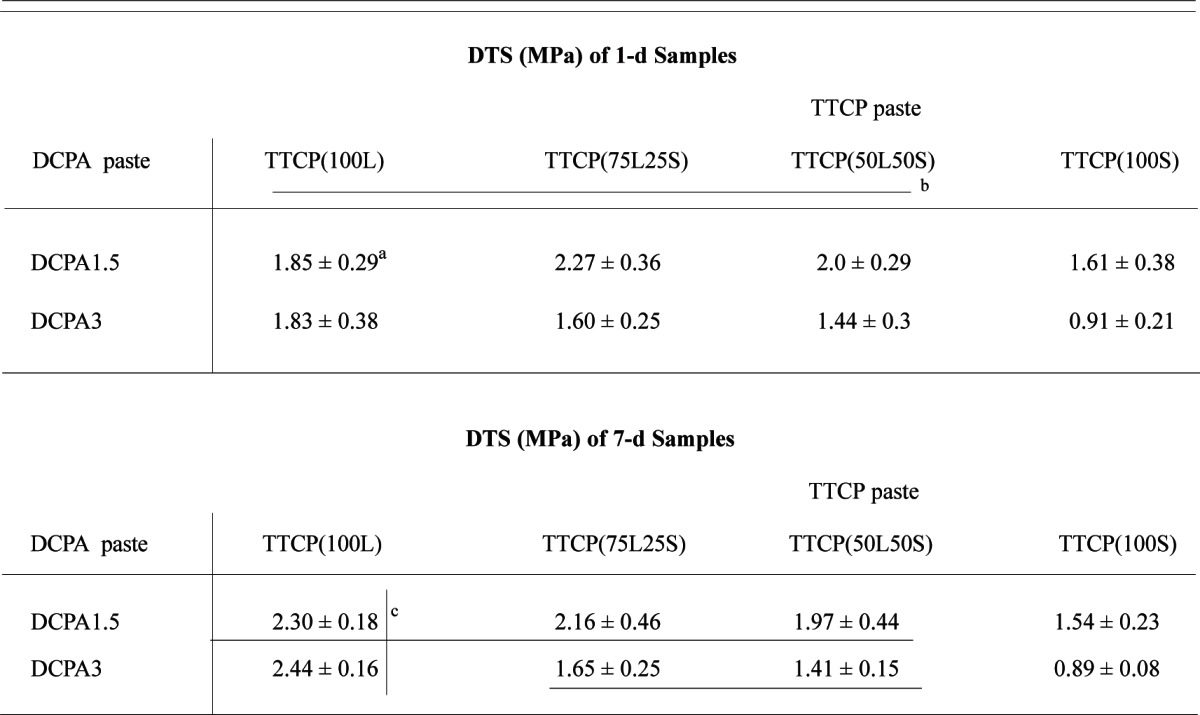

a Mean ± s.d. (n = 5)

b Groups connected by a line on the margins are not significantly different (p > 0.05)

c Groups connected by a line are not significantly different (p > 0.05)

## Footnotes

1 Certain commercial materials and equipment are identified in this paper to specify the experimental procedure. In no instance does such identification imply recommendation or endorsement by the National Institutes of Health or American Dental Association Foundation or National Institute of Standards and Technology or Tokyo Medical and Dental University or that the material or equipment identified is necessarily the best available for the purpose.
